# Two Novel Mutations Identified in an African-American Child with Chediak-Higashi Syndrome

**DOI:** 10.1155/2010/967535

**Published:** 2010-03-24

**Authors:** Kerry Morrone, Yanhua Wang, Marjan Huizing, Elie Sutton, James G. White, William A. Gahl, Karen Moody

**Affiliations:** ^1^Children's Hospital at Montefiore, Albert Einstein College of Medicine, Bronx, NY 10467, USA; ^2^Department of Pathology, Montefiore Medical Center, Albert Einstein College of Medicine, Bronx, NY 10467, USA; ^3^Section on Human Biochemical Genetics, Medical Genetics Branch, National Human Genome Research Institute, NIH, Bethesda, MD 20814, USA; ^4^Department of Laboratory Medicine, University of Minnesota School of Medicine, Minneapolis, MN 55455, USA

## Abstract

*Background.* Chediak-Higashi syndrome (CHS) is a rare, autosomal recessive disorder characterized by oculocutaneous albinism, immunodeficiency, coagulopathy and late-onset, progressive neurological dysfunction. It also has an “accelerated phase” characterized by hemophagocytic lymphohistiocytosis (HLH). The disease is caused by mutations in the *CHS1/LYST* gene located on chromosome 1, which affects lysosome morphology and function. We report the case of an African-American child with CHS in Case. This 16-month old African-American girl presented with fever and lethargy. The proband had pale skin compared to her parents, with light brown eyes, silvery hair and massive hepatosplenomegaly. Her laboratory evaluation was remarkable for pancytopenia, high serum ferritin and an elevated LDH. Bone marrow aspirate revealed large inclusions in granulocytes and erythrophagocytosis consistent with HLH. Genetic evaluation revealed two novel nonsense mutations in the *CHS1* gene: c.3622C > T (p.Q1208X) and c.11002G > T (p.E3668X). *Conclusions.* Our patient is one of the few cases of CHS reported in the African American population. We identified 2 nonsense mutations in the *CHS1* gene, the first mutation analysis published of an African-American child with Chediak-Higashi Syndrome. These two mutations predict a severe phenotype and thus identification of these mutations has an important clinical significance in CHS.

## 1. Introduction

Chediak-Higashi Syndrome (CHS) (MIM: 214500) is a rare autosomal recessive immune disease characterized by oculocutaneous albinism, a predisposition for infections, coagulopathies, neurological dysfunction, and large granules in many cell types [1–3]. CHS is the result of a genetic defect in the human *CHS1/LYST* gene (chromosome 1q421-q42.2), coding for the lysosomal trafficking regulator gene CHS1, resulting in defective formation of secretory vesicles and lysosomes [3–7]. Patients with CHS contain giant cytoplasmic inclusion bodies, large lysosomes, and lysosome-related organelles such as melanosomes. Patients have an increased risk of infection due to defects in natural killer cell activity, T-cell cytotoxicity, chemotaxis, and bactericidal killing by granulocytes and monocytes. A complication of CHS is hemophagocytic lymphohistiocytosis (HLH), which is also known as the “accelerated phase” of the disorder [5]. This phase is characterized by fever, jaundice, hepatosplenomegaly, lymphadenopathy, pancytopenia, and bleeding [8]. Ten to fifteen percent of CHS patients exhibit a milder clinical phenotype, with fewer or no severe infections and without an “accelerated phase” of the disease. Though these patients typically survive to adulthood, they are prone to developing progressive neurological dysfunction with age [6, 9]. 

The exact role of the CHS1 protein is unknown, but has been better defined through recent studies. CHS1 is thought to play a role in regulating vesicular trafficking and lysosomal and lysosome-related organelle size [3, 10, 11]. The milder phenotype of this disease occurs in patients who carry at least one missense mutation causing a partially functioning CHD1. Individuals with the more severe CHS phenotype usually have null mutant *CHS1* alleles, predicting the absence of the CHD1 protein. The greater the impairment of the lysosomal function, the more susceptible an individual is to infections [3, 5, 6]. 

The “accelerated phase” is the most life threatening clinical feature of CHS, due to the high risk of infection and hemorrhage [5, 8, 12]. Treatments with etoposide and corticosteroids have resulted in transient remissions, but relapses are frequent and bone marrow transplants have been the only effective cure [8, 12]. The determining factor for remission after bone marrow transplantation is disease status at transplantation, and transplantation is most successful when a patient with CHS is not in the “accelerated phase” [12]. 

The majority of patients with CHS are generally of Caucasian or Japanese descent [5]. So far, not more than 5 cases of Chediak-Higashi syndrome in African-American patients have been described, and to our knowledge, no mutation analysis in these patients was reported [13–18]. Here we present a detailed clinical presentation of an African-American child with CHS, born to nonconsanginous parents. In addition, we describe the *CHS1* mutation analysis identifying two novel nonsense mutations in her *CHS1* gene.

## 2. Case Presentation

A sixteen-month-old African-American girl, presented to the Children's Hospital at Montefiore emergency room with a history of fever, decreased activity, poor feeding, increased sleepiness and irritability. She had no nausea, vomiting, diarrhea, urinary symptoms, cough or runny nose. The child was previously healthy, born full term without complications to a 24-year-old G2P1001 mother. On arrival to the emergency room she was febrile, tachypneic, tachycardic, and clinically in respiratory distress.

On examination she was noted to have silvery hair, blue eyes, and pale skin. Her eyelids were edematous, but she otherwise had a normal ear, nose, and throat exam. The heart and lung examination showed sinus tachycardia and tachypnea, but were otherwise within normal limits. The patient had a protuberant abdomen with massive hepatosplenomegaly. She had meningismus, but otherwise had a grossly normal neurologic exam and normal CSF studies and culture.

**Figure 1:**
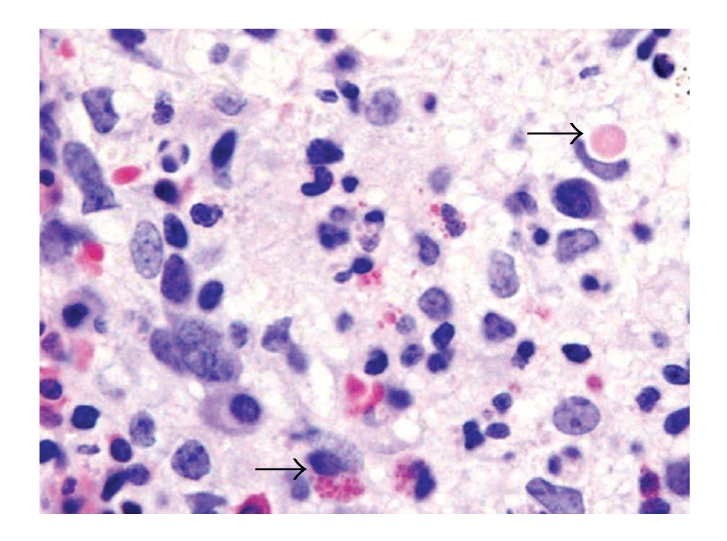
Wright stained bone marrow aspirate smear of the patient with Chediak-Higashi. The arrows in the upper right-hand side point to histiocytes engulfing intact red blood cells. The arrows in the lower left indicate fragmented red blood cells (hemophagocytosis).

**Figure 2:**
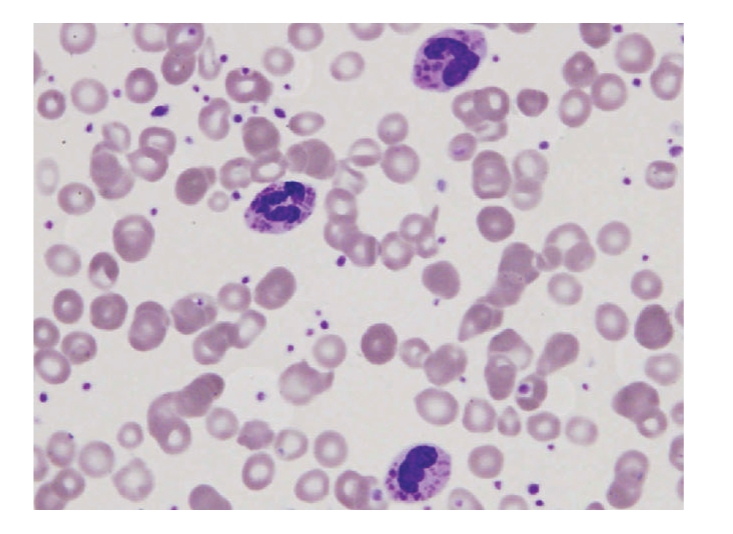
Peripheral blood smear of our patient with Chediak-Higashi depicting three polymorphonucelar leucocytes with inclusion bodies. These findings are typical in Chediak-Higashi syndrome.

**Figure 3:**
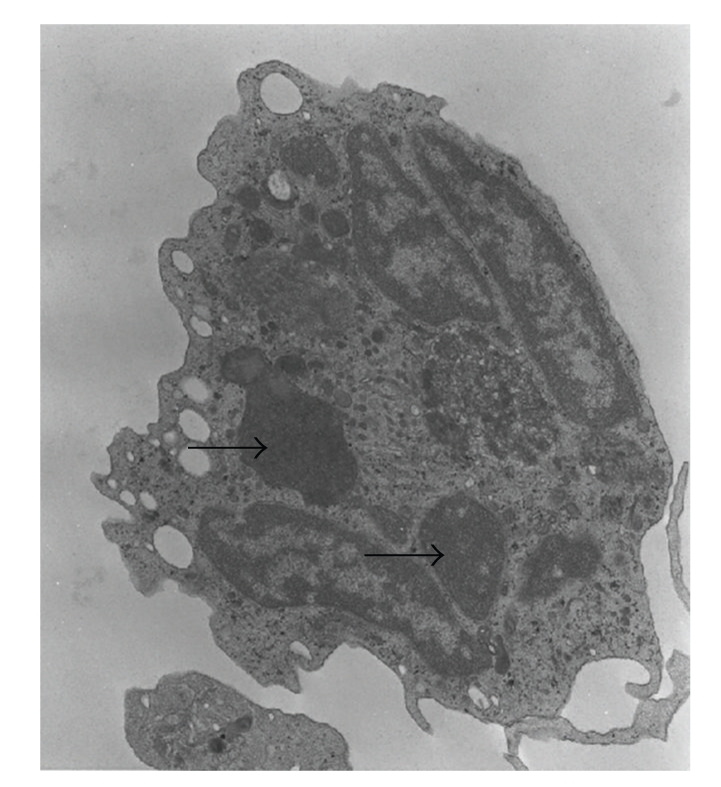
Electron microscopy of a neutrophil granulocyte of our patient. The arrows show abnormal large cytoplasmic inclusions.

**Figure 4:**
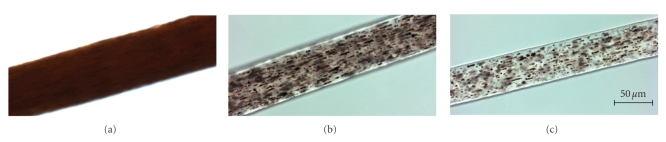
Hair shaft samples under high powered light microscopy. (a) African-American control hair sample, demonstrating evenly distributed pigment in the hairshaft. (b) Hair sample of a previously published severely affected Chediak-Higashi syndrome patient [19], demonstrating an atypical granular distribution of “pigmented clumps” in the hair shaft. (c) Hair sample of our African-American patient with Chediak-Higashi syndrome, demonstrating a similar atypical granular pigmentation pattern as the CHS patient in (b). All images were taken at the same settings using a 40X/1.3 oil objective on an Axiovert200M light microscope.

Initial laboratory results revealed pancytopenia, coagulopathy, metabolic acidosis, elevated LDH and normal uric acid. Our patient received a full sepsis, workup and was started on broad spectrum antibiotics for febrile neutropenia. The diagnosis of Chediak-Higashi syndrome was promptly determined after review of the peripheral blood smear and bone marrow biopsy (Figures 1 and 2). Bone marrow aspirate revealed large inclusions in myelocytes and erythrophagocytosis consistent with CHS and HLH. The bone marrow biopsy was diagnostic with sheets of histocytes with pale foamy cytoplasm, and pink granules in neutrophils both consistent with HLH in the accelerated phase. Electron microscopy revealed large inclusions in the granulocytes diagnostic of CHS (Figure 3). Interestingly this patient had platelets that were hypogranular to agranular as seen by electron microscopy, an unusual characteristic in CHS. Dermatology and genetics specialists assisted in confirmation of the diagnosis based on their clinical findings and the patient's hair analysis (Figure 4). The patient's hair analysis was consistent with other Chediak-Higashi hair samples [7, 19]. Further questioning of the parents revealed three maternal uncles had silvery hair, fair skin and frequent infections as children without any neurologic dysfunction. 

The patient received multiple transfusions including platelets, packed red blood cells, fresh frozen plasma and cryoprecipitate for her anemia, thrombocytopenia and coagulopathy. Within twenty-four hours of presentation she was given chemotherapy with etoposide, dexamethasone and cyclosporine as per protocol [20]. She had an excellent response after chemotherapy and a repeat bone marrow showed resolution of the histiocytic infiltration. Shortly after the patient was discharged she had to be readmitted for hypotensive shock and C difficile colitis. She recovered with broad spectrum antibiotics with fluid resuscitation and then was referred to another facility, but unfortunately, the patient succumbed to her disease before being able to receive a bone marrow transplant. 

## 3. Molecular Analysis

**Figure 5:**
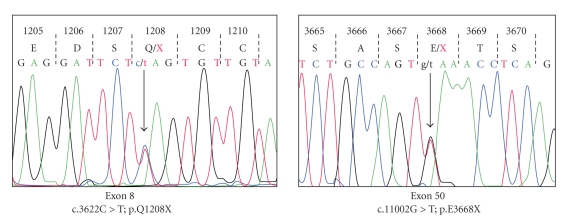
Two heterozygous nonsense mutations in the *CHD1 *gene in our patient; p.Q1208X in exon 8 and p.E3668X in exon 50.

After written consent was obtained, our patient's blood was sent for genetic testing to be done at the NIH. Genomic DNA was investigated for mutations in the *CHD1* gene (GenBank NM_000081). Each of the 53 exons and their surrounding intronic regions were PCR amplified, sequenced and analyzed, using standard methods. Two heterozygous nonsense mutations in *CHD1* were identified (Figure 5). Exon 5 displayed a c.3622C > *T* mutation, resulting in a change of a glutamine at codon 1208 to a termination codon (CAG > *T* *A* *G*; p.Q1208X). And exon 50 contained a heterozygous c.11002G > *T* mutation, resulting in a change of a glutamic acid at codon 3668 to a termination codon (GAA > *T* *A* *A*; p.E3668X). These mutations are predicted to result in nonsense mediated mRNA decay, and absence of a translated CHS1 protein. 

## 4. Discussion

 Chediak-Higashi syndrome is a rare autosomal recessive disorder characterized by varying degrees of oculocutaneous albinism, easy brusability and increased infections [3]. There is abnormal natural killer cell function with impaired chemotaxis and bactericidal activity. The underlying defects involve impaired lysosome-related organelle biogenesis [2, 6, 7, 21, 22].

 We describe a case of CHS in the black population. Her clinical picture, laboratory studies, bone marrow, and genetic evaluation confirmed CHS. She presented with a severe phenotype in the accelerated phase of CHS and had certain unusual phenotypic features such as agranular platelets. We identified 2 novel nonsense mutations in the *CHS1* gene (p.Q1208X and p.E3668X), which predict the absence of a translated CHD1 protein, along with a severe phenotype as seen in our patient. Hence identification of these mutations has an important clinical significance in predicting the phenotypic severity of patients with CHS. Furthermore, these mutations are the first *CHD1* defects described in the black population, and may serve as a source for future mutation analysis in this population.
